# Microbial succession and its correlation with the dynamics of flavor compounds involved in the fermentation of Longxi bacon

**DOI:** 10.3389/fmicb.2023.1234797

**Published:** 2023-08-31

**Authors:** Yuling Qu, Jianmin Yun, Yanhu Li, Duiyuan Ai, Wenwei Zhang

**Affiliations:** ^1^College of Food Science and Engineering, Gansu Agricultural University, Lanzhou, China; ^2^Zhuanglang County Food and Drug Inspection and Testing Centre, Pingliang, China

**Keywords:** Longxi bacon, high-throughput sequencing, microbial community, volatile flavor compounds, fatty acids, correlation analysis

## Abstract

**Introduction:**

Longxi bacon is a traditional fermented meat from Gansu province, China. The ripening process of the bacon is crucial for quality and flavor. The aim of this study was to gain deeper knowledges on the bacterial and fungal community diversity and the changes of chemical components including fatty acids and volatile compounds at different time points during the ripening of the bacon and to understand the relationship between microbial profiles and the chemical components related the bacon flavor.

**Methods:**

Bacon samples were collected from days 0, 15, 30, 60 and 90. The bacterial and fungal compositions were analyzed with next generation sequencing targeting the 16S rDNA loci for bacteria and ITS loci for fungi. The fatty acids and the volatile components were analyzed by headspace solid phase micro extraction followed by gas chromatography/mass spectrometry (HS-SPME-GC/MS).

**Results:**

We found that the abundance of bacteria in bacon was higher than that of fungi, and *Psychrobacter*, *Brochothrix*, *Phoma* and *Trichoderma* was the dominant bacon’s population. The largest contributors of volatiles were aldehydes, ketones and esters, and the main fatty acids were palmitic, oleic and linoleic acids. Pearson correlation analysis between microbial succession and key flavor substances showed that the production of Longxi bacon flavor is the result of a combination of bacteria and fungi. Ten bacteria genera and six fungi genera were determined as functional core microbiota for the flavor production based their dominance and functionality in microbial community. In addition, bacteria and fungi are involved in the oxidation and hydrolysis of fatty acids during the ripening of bacon, which also contributes to the formation of bacon flavor.

**Discussion:**

This study provides a comprehensive analysis of the key microbiota involved in shaping bacon’s distinctive flavor. Here, the results presented should provide insight into the influence of the microenvironment on the microbial community in bacon and lay a foundation for further investigations into the food ecology of bacon.

## 1. Introduction

Bacon, a traditional fermented meat product in China, is becoming increasingly popular for its unique flavor and attractive color (Huang et al., 2020). Fermentation is the most important part in the process of bacon processing, which is susceptible to multiple factors such as raw materials and environment. For example, the surface of the raw meat is exposed to the environment, and natural microbial colonization influences the fermentation process. Different geo-climatic conditions and production techniques, combined with the influence of environmental microorganisms on fermentation, allow bacon to obtain a unique aroma, flavor and texture (Lv et al., 2019a; Wang et al., 2019). Longxi pork bacon is usually processed in winter, using fresh pork belly as the raw material, and generally consists of cutting, curing (spices and 10% salt), drying and ripening. Longxi bacon has become one of the best bacon due to this unique processing method.

The product characteristics (e.g., flavor, nutritional value and texture) of fermented meat products are often greatly influenced by environmental microorganisms (Bernardi et al., 2019; Lv et al., 2019b). Abundant microbial populations are present in meat ecosystems, including *Lactobacillus*, *Leuconostoc*, *Macrococcus*, *Staphylococcus*, *Candida* and *Debaryomyces* (Asefa et al., 2009; Yi et al., 2016). Some of them can endow meat products with good flavor, anti-oxidation effects and special taste. Therefore, it is necessary to investigate the species of bacteria and fungi, and their roles they play in the production process of Longxi bacon.

The microbial community that inhabits during the ripening process of bacon plays an important role in the formation of flavor compounds, especially during fat oxidation and proteolysis. Moreover, the metabolism of microbial community in bacon determines the type and content of flavor compounds. Recently, the traditional fermentation meat products have shown that *Lactobacillus*, *Staphylococcus* and *Macrococcus* spp. can secrete proteases, lipases and 4-nitrate reductase that degrade proteins, lipids and other compounds in bacon into ketones, esters and acids with distinctive aromas (Yi et al., 2016). Additionally, *Staphylococcus carnosus* encourages the production of aldehydes like hexanal, pentanal and octanal, which enhance the flavor of fermented meat products (Engelvin et al., 2000). *Debaryomyces hansenii* produces aldehydes such as 2-methylbutyraldehyde, and 3-methylbutyraldehyde by secreting aminopeptidase, which breaks down proteins and produces free amino acids, giving the meat better flavor (Bolumar et al., 2003). *Psychrobacter* can metabolize lipids and hydrolyze amino acids (Zhang Q. et al., 2021). Considering the production process, environmental conditions and regional differences in Longxi bacon may lead to different microbial community and metabolic characteristics, which may have a significant impact on the quality and stability of Longxi bacon. Therefore, it is essential to investigate the correlation that exists between microbiota and bacon flavor, especially between the core microorganisms and characteristic flavor.

In recent years, high-throughput sequencing has been widely applied to more effectively understand the microbial community of fermented products because it transcends the limitations of traditional culture methods (Zhang et al., 2018). A large number of studies have also emerged on the correlation between microbial communities and flavor in fermented meat products, e.g., sausages (Kim et al., 2022), Jinhua ham (Wang Y. B. et al., 2021), Guizhou suan zuo rou (Wang H. et al., 2021). However, most studies on bacon flavor and microbial community succession have focused on smoked bacon (Song et al., 2022), and little research has been done on the flavor components of air-drying Longxi bacon.

In this study, the composition and dynamic changes of microbial communities in Longxi bacon ripening were characterized by MiSeq sequencing. In parallel, the changes of flavor composition during the same process were detected by chromatography, as well as the physicochemical properties. Then, the interrelationships between microbiota and physicochemical properties and flavor composition in Longxi bacon using redundancy analysis (RDA) and Pearson’s correlation analysis and reveal the functional microbial community related to the formation of the characteristic flavor compounds in bacon products. This study will enhance our understanding of the fermentation mechanisms involved in bacon production and lay a foundation for further investigation into the food ecology of bacon.

## 2. Materials and methods

### 2.1. Sample collection

**Figure 1:**
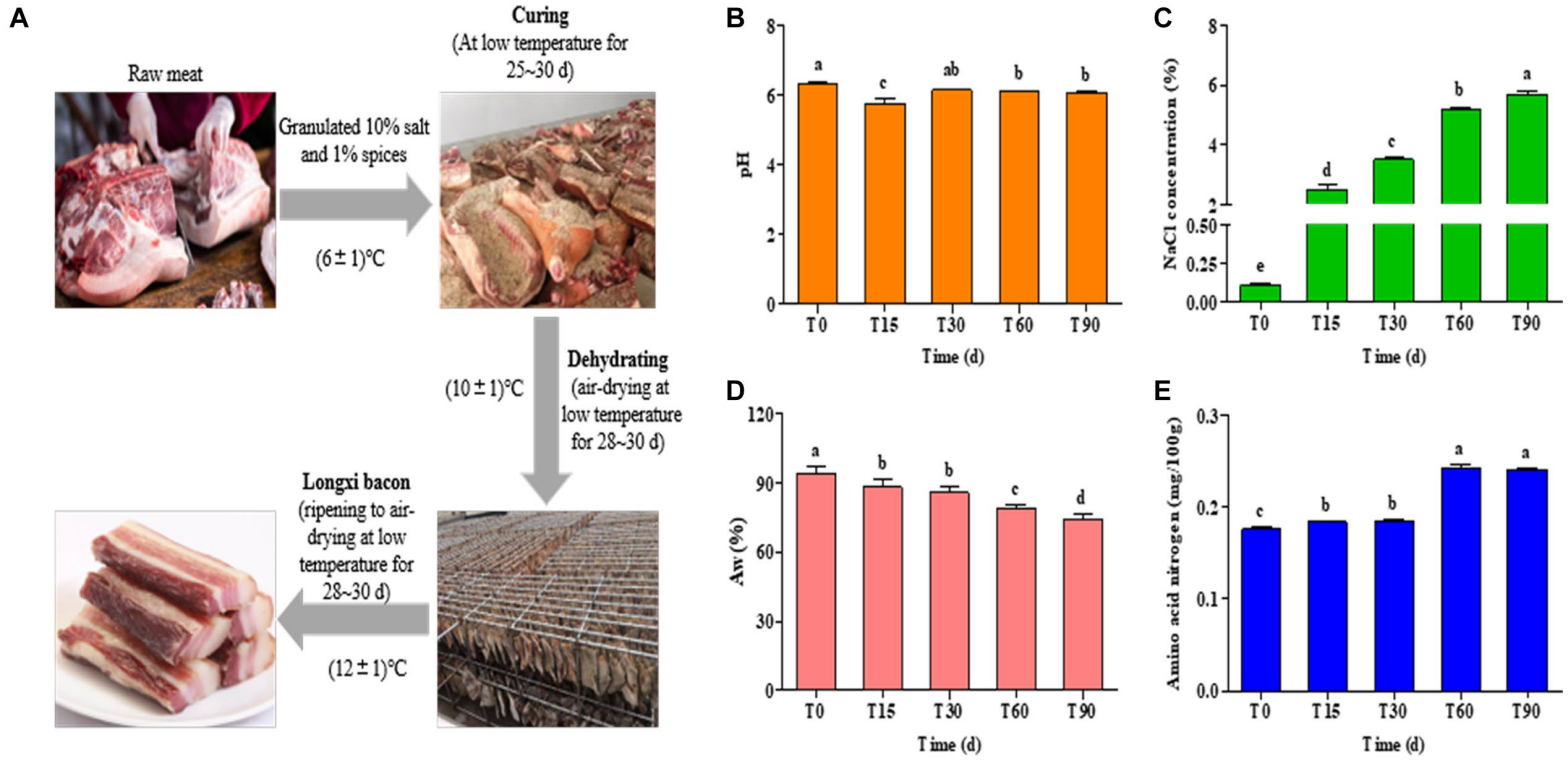
**(A)** Process diagram for traditional Longxi bacon fermentation. **(B–E)** Changes in the physiochemical indexes during Longxi bacon fermentation.

All samples were collected from a traditional Longxi bacon processing workshop located in Longxi, Gansu province, China (34.98 N, 104.61 E). According to the processing technology of bacon, three bacon samples were randomly collected at 0, 15, 30, 60 and 90 days (Figure 1A and Supplementary Table S1). The bacon samples were packed in sterile bags and transported to the laboratory on ice. In sampling, the surface of bacon tissues (upper surface and lower surface, 10 × 10 cm^2^) and the inner part (a 2 cm layer of tissue under the skin) were sliced using a sterilized scalpel, and then shred with scissors and mixed evenly, packed into a sterile sampling tube. Then, newly collected samples were directly used for physicochemical analysis and the samples for high-throughput sequencing were stored at −80°C. Each experiment was repeated three times.

### 2.2. Physicochemical indexes analysis

The pH value of the bacon was measured using a pH meter (P611, Shanghai, China). The water activity (Aw) was determined with a Fast-lab water activity meter (HD-3A, Beijing, China). The sodium chloride (NaCl) concentration was determined by titrimetric analysis (Guo et al., 2016). The amino acid nitrogen content, peroxide value (POV) and acid value were measured according to Chinese Standards GB 5009.235-2016, GB 5009.227-2016, and GB 5009.229-2016, respectively.

### 2.3. DNA extraction and Hiseq sequencing

DNA was extracted using the DNeasy PowerSoil Kit (Qiagen, Inc., Netherlands) according to the manufacturer’s instructions. The concentration and quality of the extracted DNA were measured using a NanoDrop ND-1000 spectrophotometer (Thermo Fisher Scientific, Waltham, MA, United States) and 1% agarose gel electrophoresis, respectively. For bacteria, the V4–V5 region of the 16S rDNA was amplified with primers 515F (5′-GTGCCAGCMGCCGCGGTAA-3′) and the reverse primer 907R (5′-CCGTCAATTCMTTTRAGTTT-3′). For fungi, the ITS1 region of 18S rDNA was amplified using primers ITS5F (5′-GGAAGTAAAAGTCGTAACAAGG-3′) and ITS1R (5′-GCTGCGTTCTTCATCGATGC-3′). PCR amplicons were purified with AxyPrep DNA Gel Extraction Kit (Axygen Biosciences, Sigma-Aldrich, CA, United States) following the manufacturer’s instructions. Subsequently, the next generation sequencing library preparation and Illumina MiSeq sequencing were conducted at the Shanghai Personal Biotechnology Co. Ltd. (Shanghai, China).

The paired-end reads from the DNA sequences were assembled with FLASH (Mago and Salzberg, 2011). QIIME (v1.8.0) software was used to process the sequencing data (Caporaso et al., 2010). The remaining high-quality sequences were clustered into operational taxonomic units (OTUs) at 97% sequence identity, by UCLUST (Edgar et al., 2011). A representative sequence was selected from each OTU according to the default parameters. The relative abundance (%) of the individual taxa within each community was estimated by comparison of the number of sequences assigned to a specific taxon to the number of total sequences obtained for that sample. Alpha diversity analysis, which included Good’s coverage, Simpson, Chao1, ACE, and Shannon, were calculated using MOTHUR software (V.1.31.2, http://www.mothur.org).

### 2.4. Extraction and determination of volatile components from bacon using HS-SPME-GC/MS

The HS-SPME-GC-MS method was used for the extraction and determination of the volatile components (Guo et al., 2021). Briefly, 10 g of bacon samples and 2 g of NaCl were placed into 20 mL headspace vials with silicon sealing gaskets and heated in a 70°C water bath for 10 min. Then, the headspace extraction head was inserted into the vial and the fiber head was pushed out for the volatile compounds’ headspace extraction. The extraction time was 30 min. Following extraction, the fiber was injected into the GC inlet (245°C) and desorbed for 10 min. Separation of the volatile components was performed on a DB-WAX column (30 m long × 0.25 mm i.d. × 0.25 μm film thickness) (Agilent, Santa Clara, CA, United States). The programmed sequence for the column involved an initial temperature of 30°C for 1 min, followed by an increase of 5°C/min to 180°C, at which temperature it remained for 3 min. Thereafter, the temperature was raised to 200°C at 4°C/min and held at this stage for 6 min. The temperature was then increased to 245°C at 20°C/min and held for 15 min. The helium flow rate was 1.0 mL/min. The MS working conditions were as follows: ionization energy at 70 eV, scan range at 40–600 m/z, and ion source temperature at 230°C. Volatile compounds were identified using NIST 14 (National Institute of Standards and Technology, Gaithersburg, MD, United States). Finally, the relative content of the volatile substances that were identified was calculated using area normalization. 2-Octanol (CAS: 6169-06-8) was used as an internal standard for quantification analysis. The volatile compound content was calculated by comparing its area with the internal standard (substances with positive and negative matching degrees >800 were reported).

### 2.5. Evaluation of key volatile substances

The key volatile flavor substances in the sample were determined by relative odor activity value (ROAV) analysis. Before this, it is necessary to determine the volatile components that contribute the most to the flavor of the sample. In view of this, the concept of odor activity value (OAV) was introduced as follows:


(1) OAV=Mass concentration(C)/Odor threshold(T)

Here, ROAV_max_ = 100 is defined as the volatile component that contributes the most to the sample flavor, and the ROAV values of other volatile components were calculated according to Eq. (2):


(2) $$\mathrm{ROAV}\approx C_{i}/C_{\max}\times T_{\max}/T_{i}\times 100$$

where *C_i_* and *C*_max_ represent the relative content (%) of each volatile component and the relative content (%) of the volatile component with the largest contribution to the overall flavor of the sample, respectively. *T_i_* and *T*_max_ represent the odor threshold (μg/L) of each volatile component and the odor threshold (μg/L) of the volatile components that contributes the most to the overall flavor of the sample, respectively.

### 2.6. Analysis of free fatty acids content

Analysis of fatty acid content was performed as described previously (De Pasquale et al., 2014). Briefly, 1 g bacon sample was put into a test tube with a plug, 0.7 mL 10 M KOH and 5.3 mL anhydrous methanol were added and placed in a constant water bath at 55°C for 1.5 h, shaking at 20 min intervals. After the water bath was cooled to room temperature, 0.58 mL 12 M H_2_SO_4_ was added and the water bath immersion was continued for 1.5 h with shaking every 20 min. After cooling, 3 mL hexane was added, mixed until homogeneous and transferred to a 50 mL centrifuge tube and centrifuged for 5 min at 3000 × *g* and the supernatant was collected. Finally, the collected supernatant was filtered with a 0.22 μm semipermeable membrane and then subjected to GC-MS analysis (Agilent, Santa Clara, CA, United States). The GC-MS conditions were as follows. The carrier gas was helium, the capillary column was a VF-WAX (30 m long × 0.25 mm i.d., 0.25 μm film thickness) and the flow velocity was 1.0 mL/min. The oven temperature was 30°C during the first 1 min and was then increased at 5°C/min to 180°C. After 3 min, temperature was increased to 200°C at 4°C/min. Finally, the temperature was increased to 245°C at 20°C/min and was held for 15 min. The inlet temperature was 250°C, without diversion sampling. The MS working conditions were as follows: ionization energy at 70 eV, scan range at 40–400 m/z, parent ion m/z 265, transmission line temperature at 250°C, activation voltage at 1.0 V and ion source temperature at 230°C. Volatile compounds were identified using NIST 14 (National Institute of Standards and Technology, Gaithersburg, MD, United States). The MS data were determined using the NIST library, and the relative content of each component was calculated by the peak area normalization method.

### 2.7. Sensory evaluation

The sensory analysis was performed by ten panelists (5 males and 5 females, aged 23–30 years and healthy) who were experienced and engaged in food flavor research for sensory evaluation. Finally, the quantitative descriptive analysis was performed in triplicate by panelists, and scores for each sample in one increment, from 14 to 20 on the basis of a 7-point scale (14, none; 17.5, moderate; and 20, very strong).

### 2.8. Statistical analyses

All physicochemical experiments in this study were repeated three times to ensure accuracy and the results were shown as the mean ± standard deviations (SD). SPSS 19.0 was used for multiple comparison significance analysis, and *p* < 0.05 was considered significant. The bar charts were drawn with Origin 9.0, The RDA was preformed using Canoco (version 4.5, Biometris Plant Research International, Wageningen University, Wageningen, The Netherlands). Principal component analysis (PCA) was performed with SIMCA-P software (version 11.5, Umetrics, Umeå, Sweden). Spearman correlation analysis was performed using R (version 2.15.3; The R Foundation for Statistical Computing, Vienna, Austria).

## 3. Results

### 3.1. Changes in the physicochemical properties during fermentation of Longxi bacon

The physicochemical properties of Longxi bacon are determined primarily by the production procedures and fermentation time, which affected the microbial community succession in the food microenvironment. Changes in the pH, Aw, NaCl and amino acid nitrogen of the bacon during fermentation are presented in Figure 1. The pH of the bacon remained between 6.07 and 6.32, with only the pH of T15 being 5.76 (Figure 1B), which was significantly lower than other stages (*p* < 0.05), which might be caused by microbial activity. In general, the growth of lactic acid bacteria and the formation of organic acids can directly lead to a decrease in pH (Wang et al., 2018).

Salt is the main seasoning in the production of bacon, which has both a preservation effect and inhibits the growth of spoilage bacteria and fungi, improving the quality of the bacon and extending its storage life. As seen in Figure 1C, the NaCl concentration of the bacon samples increased significantly with the extension of processing time (*p* < 0.05). In this study, the initial NaCl concentration of the raw meat was 0.11, and the final NaCl concentration increased by 50.73% after 90 days. This result could be explained by the presence of a critical mass of salt used in bacon preparation (Nachtigall et al., 2019). It could also be said that the high final NaCl concentration of the bacon was related to the amount of salt used in its preparation.

The protein, lipid oxidation and microbial growth of the bacon were all affected by the Aw (Ojagh et al., 2010). As shown in Figure 1D, the Aw gradually decreased (*p* < 0.05) with an increase in the processing time, which was consistent with the increase of NaCl concentration. The Aw of the bacon decreased by 21.13% from T0 to T90, which may be due to osmotic dehydration caused by salt addition (Gomez et al., 2015), and the air-drying of meat was also a dehydration process.

Protein hydrolysis is an important pathway for bacon flavor formation. From Figure 1E, amino acid nitrogen showed a gradual increasing trend in the processing and the trend in increase was obvious in the late-curing stage. The content of the amino acid nitrogen in the raw meat was 0.18 mg/100 g, and increased to 0.24 mg/100 g in ripened bacon (*p* < 0.05), which was an increase of 33.33%. The increase in amino acid nitrogen content may be related to the growth and metabolism of microorganisms, which can secrete abundant enzymes to accelerate protein hydrolysis to produce amino acid nitrogen, which also contributes to the formation of the flavor of the Longxi bacon.

### 3.2. Microbial richness and alpha diversity

The sequences of the 16S rDNA and ITS loci were used to characterize the metabolically active microorganisms. No significant differences were found between the three replicates (*p* < 0.05). After quality filtering and chimera removal, 186,866 high-quality 16S rRNA gene sequences and 212,334 high-quality ITS2 region sequences were identified from the bacon samples. These sequence reads were clustered into 1,890 OTUs at a 97% similarity level per sample, of which 580 OTUs belonged to the bacteria, and 1,310 OTUs were attributed to the fungi. The indices describing alpha diversity can be found in Supplementary Table S2. The goods coverage ranged from 0.978 to 0.999 for all the samples, indicating a satisfactory description of the microbial diversity with the data coverage. The highest values of alpha diversity indices, such as Shannon index, Simpson, Chao1 richness and ACE were found for bacteria, indicating that greater diversity in bacteria.

The Venn diagrams visualizes the similarity and difference of microbial diversity in bacon at the different fermentation stages (Supplementary Figures S1A,B). The OTUs results showed that the number of common OTUs for bacteria in all samples during the production was 195. The unique OTUs for each period from T0 to T90 numbered 205, 339, 259, 204 and 364, respectively. Meanwhile, there were 53 common OTUs for fungi, and the unique OTUs for each period from T0 to T90 numbered 58, 60, 58, 35 and 83, respectively.

### 3.3. Succession analysis of microbial community in Longxi bacon at different stages

**Figure 2:**
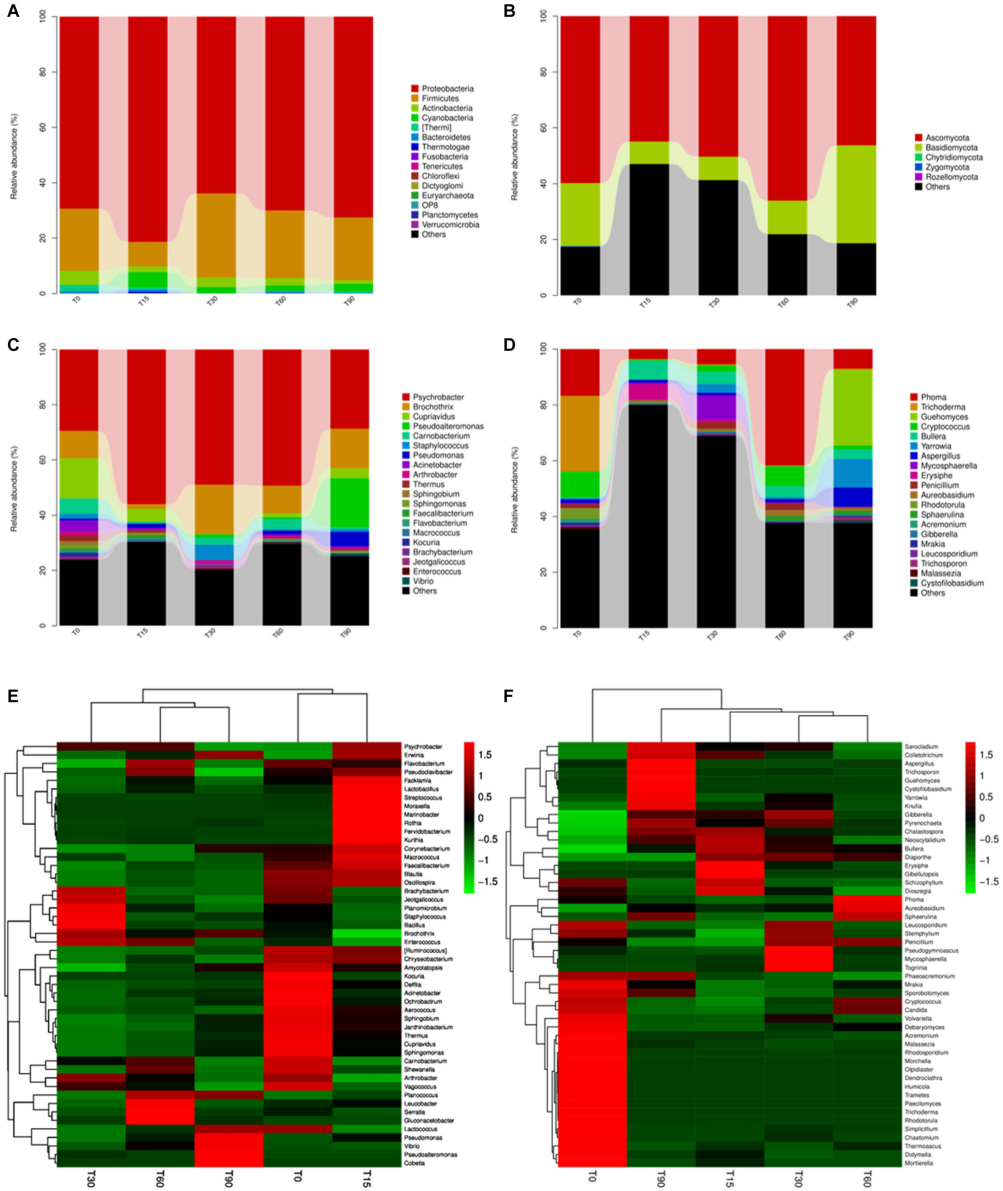
Variation in the microbial community structures at different fermentation stages. The relative abundance of bacteria at the **(A)** phylum and **(C)** genus levels. The relative abundance of fungi at the **(B)** phylum and **(D)** genus levels. Heatmap of community composition at the genus level for combined cluster analysis of **(E)** bacteria and **(F)** fungi. Each bar represents the relative abundance of each sample. Each color represents a particular phylum or genus.

The relative abundance of bacterial and fungal community percentages at the phylum and genus level for Longxi bacon at different stages are shown in Figure 2; Supplementary Figure S1C. Most bacterial communities in Figure 2A are members of the four phyla, including Actinobacteria, Firmicutes, Cyanobacteria and Proteobacteria. Among them, Proteobacteria was the dominant phylum throughout the fermentation, followed by Firmicutes. At the bacterial genus level (Figure 2C), *Psychrobacter* was the dominant genus, with the highest abundance of 56% at T15. This genus has frequently been found in cryopreserved seafood and meat, and proliferates well at low temperatures (Zhang Q. et al., 2021). *Brochothrix*, *Pseudoalteromonas*, *Cupriavidus* and *Moraxellaceae* followed. Notably, *Pseudoalteromonas* (17.7% at T90), *Staphylococcus* (5.6% at T30), *Lactobacillus* (8.5% at T60 and 5.3% at T90) were present in the later stage of fermentation, which may have provided the bacon with its unique flavor (Song et al., 2022).

As shown in Figure 2B; Supplementary Figure S1D, most fungal communities are members of Ascomycota and Basidiomycota, and their abundance values were 66.1% and 35.1%, respectively. At the fungal genus level (Figure 2D), its dominant fungal genera were primarily *Phoma*, *Trichoderma*, *Guehomyces* and *Cryptococcus* with percentages that comprised 41.7%, 27.1%, 27.1%, and 9.8%, respectively. Significant microbial succession was also observed in fungi genera, such as the abundance percentage of *Trichoderma* decreased from 27.1% at T0 to 0.5% at T90, the abundance percentage of *Cryptococcus* decreased from 9.2% at T0 to 1.4% at T90. *Guehomyces* increased from 0.1% at T0 to 27.1% at T90. It was reported to produce more flavoring substances than other yeasts owing to their production of branched-chain amino acids (BCAAs) through the Ehrlich pathway (Sana et al., 2020).

To compare structural differences and similarities in microbial community composition in samples from different periods, heatmaps combined with clustering analysis (top 50) were performed (Figures 2E,F). The results of the bacterial genus showed that all samples could be divided into three different clusters, the raw meat (T0) and the mid-curing stage (T15) were grouped in one cluster, the late-curing stage (T30) was grouped in one cluster, and the air-drying stage (T60) and the ripened Longxi bacon stage (T90) were grouped in another cluster (Figure 2E). The fungal genus level showed four different clusters, except for T30 and T60, and other samples were not clustered. These findings indicated that the curing and air-drying operations during the Longxi bacon production were important factors affecting the composition of microbial community in all samples (Figure 2F).

### 3.4. Correlations between microorganisms and physiochemical properties

**Figure 3:**
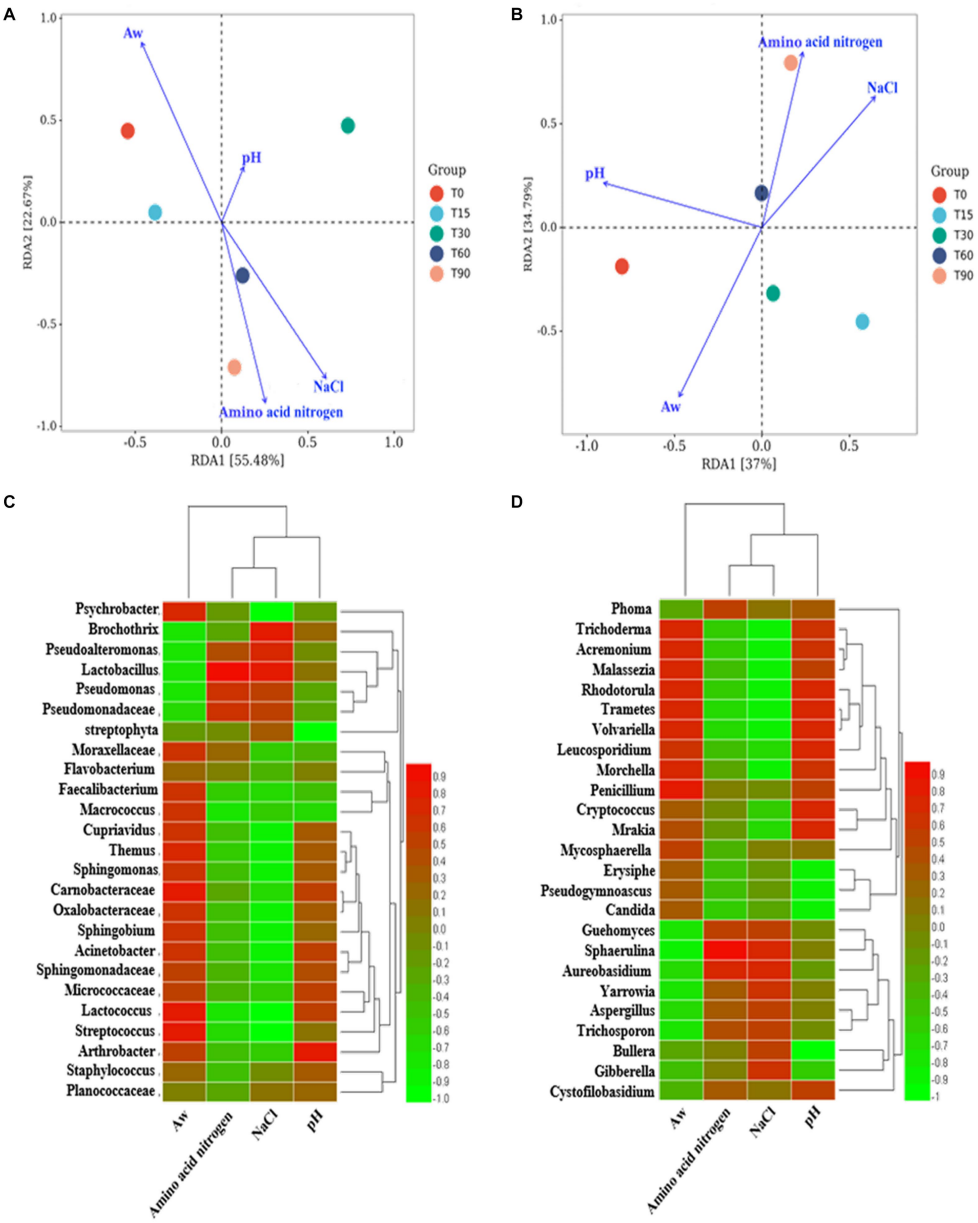
RDA analysis of the relationship between microbial community succession and physicochemical factors. Correlation analysis between the microenvironment and specific microbial genera: **(A,C)** bacteria genera, **(B,D)** fungi genera.

The factors crucial in shaping the variation in the composition of the microbial community were identified by analyzing the association between the succession of microbial community and physicochemical factors, including pH, Aw, NaCl and amino acid nitrogen, using RDA (Figure 3A). In terms of bacteria communities, the arrows in Figure 3A indicated that the most important physicochemical factor shaping the microbial succession was Aw (*p* < 0.05, *r*^2^ = 0.869), followed by NaCl (*p* < 0.05, *r*^2^ = 0.769), amino acid nitrogen (*p* < 0.05, *r*^2^ = 0.673), whereas pH (*p* > 0.05, *r*^2^ = 0.374) had little influence on the bacterial community in the different stages. For fungi, the arrows in Figure 3B indicate that Aw (*p* < 0.05, *r*^2^ = 0.816) had the greatest influence on the bacon microbiota. Therefore, it was considered that Aw was the main environmental factor that drove the variation in the fungal community in bacon. NaCl (*p* < 0.05, *r*^2^ = 0.772), amino acid nitrogen (*p* < 0.05, *r*^2^ = 0.770) and pH (*p* < 0.05, *r*^2^ = 0.760) followed.

Spearman correlation analysis was used to further illustrate the influence of the microenvironment on the abundance of specific microbial genera. As shown in Figure 3C, a large number of bacterial genera were positively correlated with Aw, negatively correlated with NaCl and amino acid nitrogen and not significantly correlated with pH. For example, the dominant genera *Psychrobacter*, *Cupriavidus* and *Carnobacterium* (*p* < 0.05). were positively correlated with Aw, and *Psychrobacter* (*p* < 0.01), *Cupriavidus* (*p* < 0.05) and *Carnobacterium* were negatively correlated with NaCl. As shown in Figure 3D, Aw/pH was positively correlated with many fungal genera (*p* < 0.05), such as Aw was positively correlated with *Phoma* and *Guehomyces*. While NaCl/amino acid nitrogen was negatively correlated with most fungi (*p* < 0.05), such as NaCl was negatively correlated with *Trichoderma* and *Cryptococcus*.

### 3.5. Changes of volatile compounds in Longxi bacon

**Figure 4:**
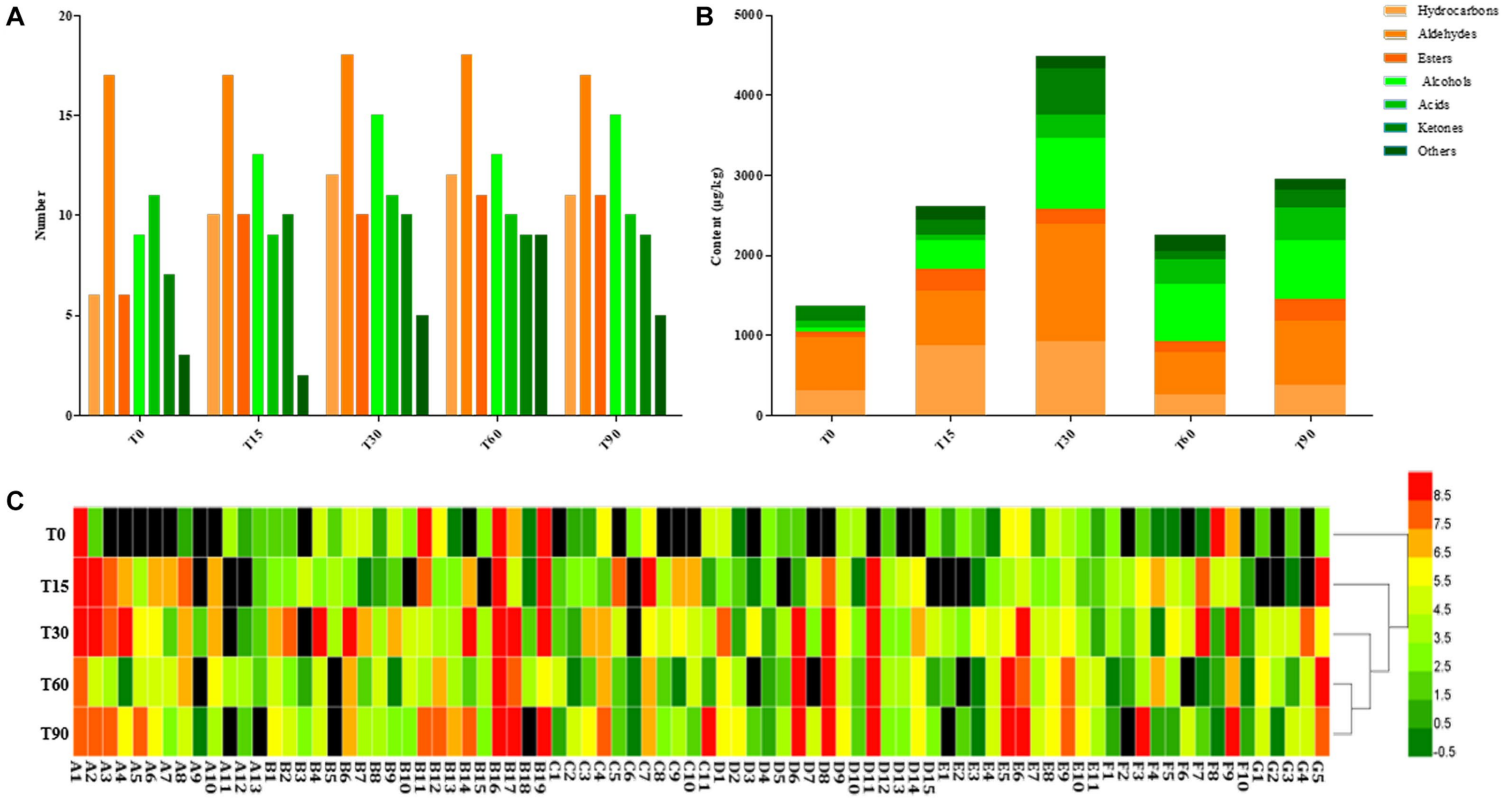
Changes in volatile components during bacon fermentation. **(A)** Numbers of volatile compound types; **(B)** Content of volatile compounds; **(C)** Heatmap of volatile components content, red: high concentration, grass green: medium concentration, and dark green: low concentration.

Most volatile compounds in meat products are formed through the smoking process, microbial metabolism and the degradation of lipids and proteins (Nachtigall et al., 2019; Gan et al., 2021). A total of 84 volatile substances were detected during the fermentation, consisting of 13 hydrocarbons, 19 aldehydes, 11 esters, 15 alcohols, 11 acids, 10 ketones and 5 others (Supplementary Table S3). From Figures 4A,B, with the extension of fermentation time, the types of volatile compounds identified from bacon increased from 59 at the initial stage to 78 after 90 days, and their content was highest at 4485.46 μg/kg at the mid-curing period. Furthermore, aldehydes and alcohols were the most abundant aroma components in the fermentation process of bacon, followed by hydrocarbons, ketones, acids and esters.

The heatmap showed that there were significant differences in the composition and content of the volatile flavor compounds in the samples from different periods (*p* < 0.05), with a larger proportion of yellow and red and less black in T30, T60 and T90, indicating that volatile flavor compounds are mainly formed in the middle and late stages of bacon production (Figure 4C). The cluster analysis showed that T30 clustered with T60 and T90 into one category, indicating that the composition and content of the volatile flavor compounds were similar in the late curing, the mid-air-drying and the ripened stages.

#### 3.5.1. Determination of key flavor compounds in Longxi bacon based on ROAV analysis

The higher the ROAV value of the volatile component, the greater the contribution to the overall flavor of the sample. Generally, the ROAV values of the volatile components between 1 and 100 are considered as the key flavor compounds in the samples, and components with ROAV values between 0.1 and 1 are considered to have an important modifying effect on the overall flavor of the samples (Xie et al., 2018). The ROAV analysis in this study showed that there were 22 volatile components with ROAV values between 1 and 100, and 7 volatile components with ROAV values between 0.1 and 1 for the samples (Table 1).

**Table 1:** Determination of volatile flavor substances during the production of Longxi bacon.

No.	Volatile compounds	Relative abundance (%)					Threshold(μg/kg)	ROAV
		T0	T15	T30	T60	T90		
S1	3-Carene	N.D.	5.13	0.30	1.43	0.57	44.00	0.272
S2	D-Limonene	0.29	29.82	15.95	2.00	9.47	10.00	7.409
S3	α-Pinene	N.D.	4.98	2.19	1.91	2.13	200.00	0.090
S4	β-Pinene	N.D.	1.10	3.10	1.91	9.35	140.00	0.178
S5	Styrene	32.38	29.16	39.97	10.85	7.75	47.00	3.291
S6	Laurylenes	N.D.	7.50	7.20	1.05	6.18	13.00	2.716
S7	(E,E)-2,4-nonadienal	0.41	0.50	8.37	0.64	1.66	0.90	16.570
S8	2,4-Decadienal	1.72	0.48	11.56	1.78	1.56	0.70	31.460
S9	2-Heptenal	2.09	0.67	15.14	6.75	3.85	13.00	2.823
S10	2-Hexenal	0.15	0.18	0.70	1.81	0.99	14.00	0.352
S11	2-Nonenal	1.65	0.30	3.37	0.11	0.70	0.10	78.944
S12	2-Octenal	20.66	6.74	1.81	1.16	6.20	3.00	15.699
S13	3-Methylbutyraldehyde	3.14	0.57	0.89	4.48	6.45	0.20	100.000
S14	Benzaldehyde	0.27	0.69	0.69	1.22	3.59	350.00	0.024
S15	Heptanal	0.50	N.D.	0.94	1.22	0.64	3.00	1.771
S16	Hexanal	24.37	44.65	56.28	32.05	25.44	4.50	52.312
S17	Nonyl aldehyde	3.80	1.73	9.57	8.69	15.15	1.00	50.148
S18	Octanal	16.72	10.18	15.64	2.61	11.68	0.80	91.484
S19	Ethyl caproate	2.91	0.36	4.77	4.86	7.89	5.00	5.355
S20	Ethyl caprylate	2.71	11.65	4.30	6.33	3.55	26.00	1.414
S21	Geranyl acetate	N.D.	3.73	1.40	1.79	0.42	9.00	1.313
S22	Ethyl acetate	1.33	0.19	0.69	0.20	11.37	5.00	3.549
S23	Heptanol	0.38	0.72	0.43	0.87	0.47	330.00	0.011
S24	1-Octen-3-ol	1.58	0.62	6.56	2.61	2.61	1.00	18.004
S25	2,3-Dibutanol	0.11	0.30	0.19	1.10	2.97	4500.00	0.001
S26	2-octen-1-ol	0.30	N.D.	0.93	0.32	1.21	40.00	0.111
S27	Phenethyl alcohol	1.13	1.80	2.12	2.14	2.14	86.00	0.140
S28	Linalool	N.D.	22.84	38.75	43.96	34.10	31.00	7.252
S29	Hexanol	N.D.	2.01	0.58	1.26	0.51	250.00	0.028
S30	Pinoresinol	N.D.	2.13	4.17	3.37	3.45	330.00	0.064
S31	Pentanol	0.52	N.D.	1.27	0.22	0.52	4000.00	0.001
S32	Butyric acid	0.40	0.11	2.39	0.15	0.11	240.00	0.017
S33	Heptanoic acid	0.13	0.51	1.51	2.09	1.53	3000.00	0.002
S34	Decanoic acid	2.23	0.82	2.92	13.91	14.80	10000.00	0.004
S35	Caproic acid	3.24	1.77	14.35	7.60	12.46	3000.00	0.017
S36	Nonanoic acid	0.16	0.67	1.21	0.99	1.50	3000.00	0.002
S37	Lauric acid	0.68	0.29	0.70	1.63	2.07	10,000	0.001
S38	Myristic acid	1.55	0.66	1.26	3.10	3.05	10000.00	0.001
S39	Octanoic acid	1.14	1.20	2.98	9.13	9.49	3000.00	0.010
S40	2,3-Octanedione	0.40	2.60	1.13	1.07	10.85	3.00	6.890
S41	Acetophenone	4.18	1.21	9.17	4.65	11.32	65.00	0.605
S42	2-Pentanone	0.13	4.03	0.12	4.23	0.24	2800.00	0.004
S43	2-Octanone	0.14	1.80	1.84	0.87	0.16	50.00	0.124
S44	Carvone	N.D.	1.05	0.30	0.22	N.D.	50.00	0.067
S45	Geranylacetone	0.31	0.15	0.13	0.12	0.22	60.00	0.020
S46	Dodecane	0.94	N.D.	N.D.	1.35	N.D.	2040.00	0.004
S47	p-Cresol	0.38	N.D.	1.10	2.46	2.03	18.00	0.534
S48	2-Pentylfuran	0.40	0.13	1.33	0.28	2.04	5.00	1.077
S49	Anisole	N.D.	17.33	N.D.	2.86	4.52	50.00	1.061

Among the olefins, D-limonene, styrene and lauricene were mainly identified as key flavor compounds, where the ROAV value of D-limonene (7.409) was much higher than that of styrene (3.291) and lauricene (2.716). However, considering the important influence of spice on the flavor formation of the bacon, the higher ROAV values of D-limonene (orange peel) and lauricene (myrcia) may be related to the spice addition during the curing period. Therefore, the influence of D-limonene and lauricene was removed and styrene was identified as the key hydrocarbon.

Aldehydes were mainly 3-methylbutyraldehyde, octanal, 2-nonenal, hexanal, nonanal, 2,4-decadienal, (E,E)-2,4-nonadienal, 2-octenal, 2-heptenal and heptanal, with 3-methylbutyraldehyde (100) being the aldehyde that contributed the most to the flavor formation of the bacon, followed by octanal (91.484), 2-nonenal (78.944), hexanal (52.312), nonanal (50.148), 2,4-decadienal (31.460), (E,E)-2,4-nonadienal (16.570), 2-octenal (15.699), 2-heptenal (2.823) and heptanal (1.771).

The key esters were ethyl caproate, ethyl acetate, ethyl caprylate and geranyl acetate, among which ethyl caproate (5.355) contributed the most to the flavor formation of the bacon. Among the alcohols, 1-octen-3-alcohol and linalool were the key flavor compounds, and 1-octen-3-alcohol (18.004) contributed the most to the flavor formation of the bacon. Among the ketones, 2,3-octanedione (6.890) contributed the most to the flavor of the bacon. In addition, alkanes and acids (ROAV <1) played a modifying role in bacon flavor formation. And together with olefins, aldehydes, esters, alcohols, ketones formed the main aroma body of the Longxi bacon, reflecting the unique aroma quality of the bacon.

#### 3.5.2. Determination of the main characteristic type of flavor in the production of Longxi bacon by PCA

**Figure 5:**
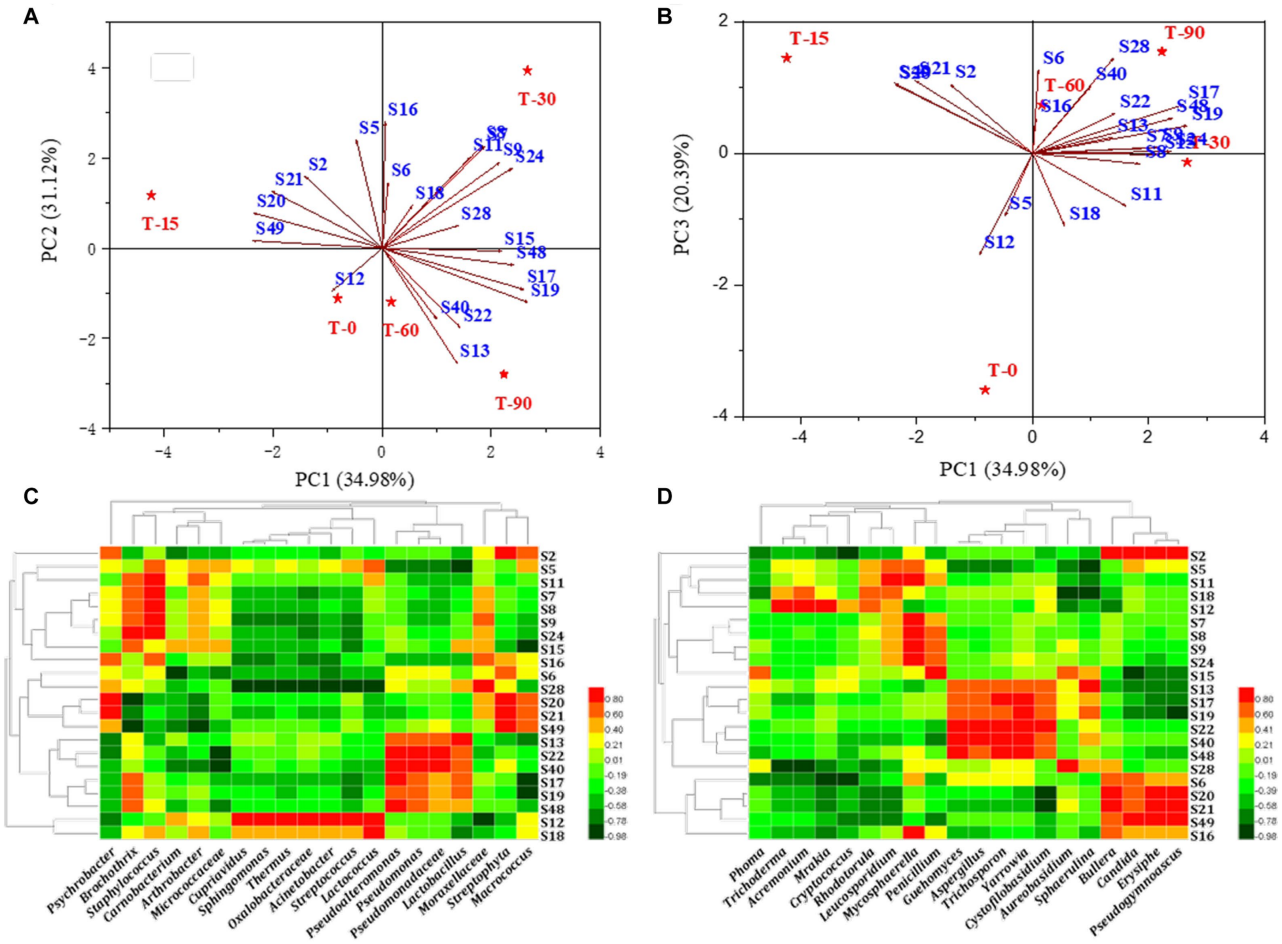
**(A,B)** Principal component loadings of key flavor substances in Longxi bacon; **(C,D)** Pearson correlation analysis of bacteria/fungi and key flavor substances. Red: positive correlation, green: negative correlation (the darker the color, the stronger the correlation).

Figures 5A,B showed that S19 (ethyl caproate), S20 (ethyl caprylate), S16 (hexanal), S13 (3-methylbutyraldehyde), S12 (2-octenal) and S28 (linalool) were the flavor substances with significant differences in contents in the samples from the different periods. Meanwhile, the principal components 1, 2 and 3 could well distinguish the samples from different periods, but T60 and T90 were always located in the same quadrant, which was similar to the clustering results, indicating that the major flavor substances were mainly formed in these two periods.

### 3.6. Correlations between microorganisms and volatile flavor compounds

Previous studies have shown that bacon flavor formation is related to microbial metabolism, we selected the bacterial and fungal genera (top 20) in the bacon processing and analyzed the correlation between them and key volatile flavor substances (ROAV >1), respectively (Figures 5C,D). Among them, 18 microorganisms (twelve bacterial and six fungal) were related to aldehydes, 14 microorganisms (seven bacterial and seven fungal) were associated with esters, 8 microorganisms (four bacterial and four fungal) were associated with ketones, 8 microorganisms (three bacterial and five fungal) were associated with hydrocarbons, 11 microorganisms (eight bacterial and three fungal) were associated with alcohols, and 3 microorganisms (two bacterial and one fungal) were related to ethers. In summary, we found that *Staphylococcus*, *Lactobacillus*, *Micrococcus*, *Cupriavidus*, *Oxalobacteraceae*, *Acinetobacter*, *Lactococcus*, *Sphingomonas*, *Streptococcus*, *Pseudogymnoascus*, *Thermus*, as well as *Acremonium*, *Aspergillus*, *Erysiphe*, *Yarrowia*, *Penicillium*, *Trichoderma* were correlated with the main flavor components S19 (ethyl caproate), S20 (ethyl caprylate), S16 (hexanal), S13 (3-methylbutyraldehyde), S28 (linalool) and S12 (2-octenal). These microorganisms are the main contributors to key flavor substances. Our results are consistent with reports indicating that they may also be involved in the formation of flavor and in the conversion of intermediate substances (Iijima et al., 2016).

### 3.7. Changes of free fatty acids during the production of Longxi bacon

**Figure 6:**
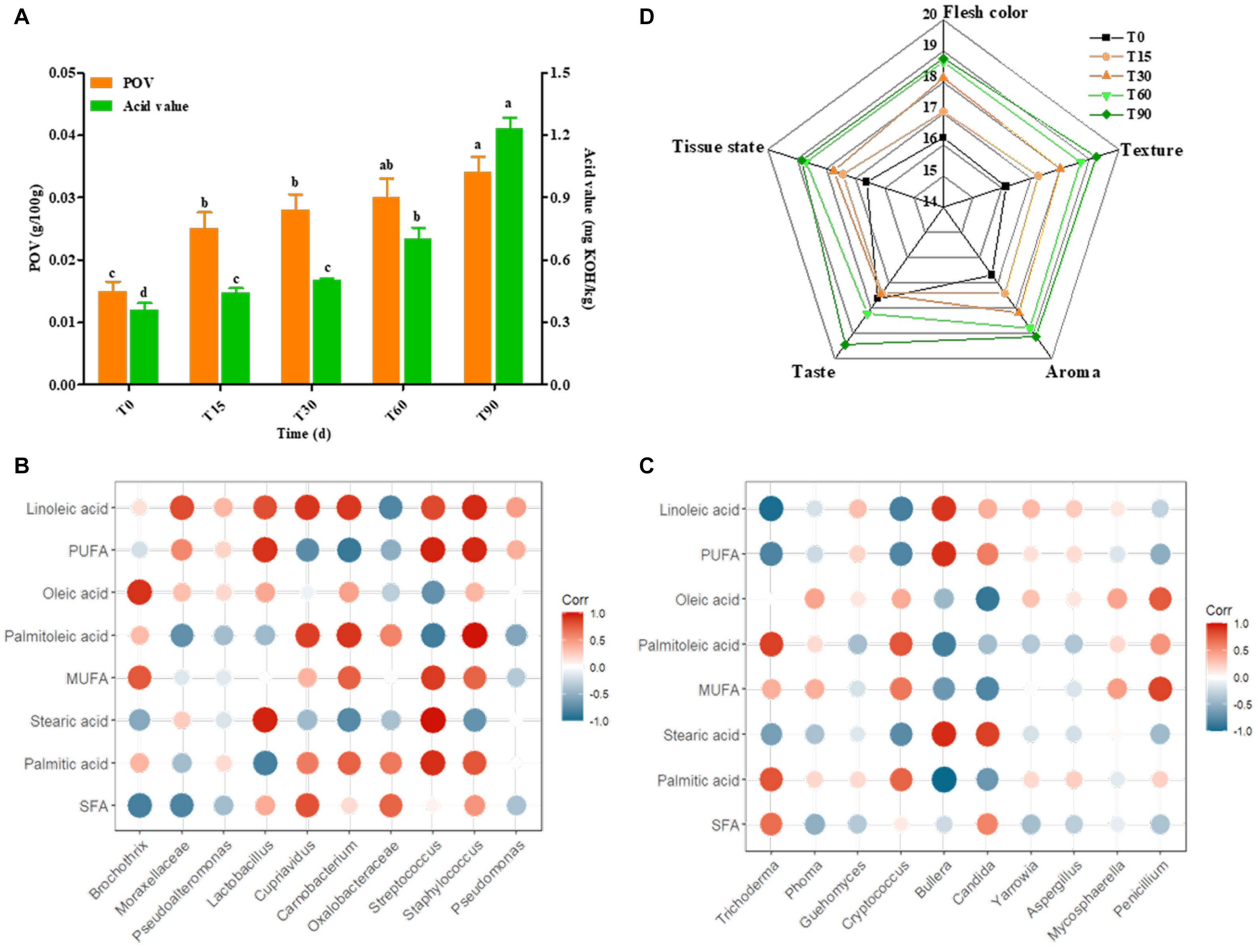
Changes in POV, acid value and sensory characteristics during bacon fermentation, and Pearson correlation analysis between the microbial community and the major fatty acids. **(A)** Changes in POV and acid value, **(B,C)** Correlation analysis between the bacterial and fungal communities and the major fatty acids, **(D)** Sensory score. Red: positive correlation, blue: negative correlation (the darker the color, the stronger the correlation).

Supplementary Table S4 showed that of the free fatty acids in bacon samples at different periods mainly included palmitic acid, oleic acid and linoleic acid. During the fermentation of bacon, the highest saturated fatty acid (SFA) content was palmitic acid, the highest monounsaturated fatty acid (MUFA) proportion was oleic acid, and the highest polyunsaturated fatty acid (PUFA) content was linoleic acid. In addition, the SFA content of the ripened meat decreased by 8.6%, and the MUFA content of the ripened meat decreased by 10.34% compared to the raw meat, which was consistent with the gradual increase in peroxide value (POV) during the processing of bacon (Figure 6A), which may be attributed to the metabolic activity of *Lactobacillus* and *Staphylococci* during the ripening of bacon, as well as the rapid accumulation of free fatty acids, which promote the rapid oxidation of fat. Moreover, the content of PUFA increased by 46% compared with the raw meat, which was consistent with the gradual increase in acid value during the processing of bacon (Figure 6A), indicating that with the extension of processing time, the fat gradually underwent hydrolysis and the fatty acids accumulated continuously leading to the increase in acid value. Nutritionists believe that the meat with a PUFA/SFA ratio >0.4 has a more balanced fatty acid composition. The results of this study showed that the ΣPUFA/ΣSFA values in bacon samples at different periods ranged from 0.291–0.505. Obviously, the fatty acid composition of Longxi bacon meets this condition.

### 3.8. Correlations between microorganisms and fatty acids

Lipid hydrolysis and oxidation are important flavor sources in bacon, and microorganisms are involved in this process (Gan et al., 2021). Therefore, we selected the main fatty acids and microorganisms (top 10) for correlation analysis in order to reveal the relationship and change pattern between fatty acids and microorganisms during the bacon production. For bacterial genera (Figure 6B), the main bacterial community associated with SFA were *Lactobacillus* and *Streptococcus*. Among them, stearic acid was significantly positively correlated with *Streptococcus* (*p* < 0.01) and *Lactobacillus* (*p* < 0.05), and palmitic acid was significantly positively correlated with *Streptococcus* (*p* < 0.05). This may be since *Lactobacillus* accelerated fat degradation by secreting lipase, which facilitated the accumulation of stearic acid, while *Streptococcus* promoted the oxidation of palmitic acid resulting in a decrease in its content in ripened bacon. The main bacterial community associated with MUFA was *Streptococcus*, where palmitoleic acid was highly significantly positively associated with *Staphylococcus* (*p* < 0.01) and significantly positively associated with *Carnobacterium* (*p* < 0.05). This may be because *Carnobacterium* and *Staphylococcus* can also secrete lipase to promote palmitoleic acid oxidation, while *Staphylococcus* acts more significantly. The bacterial community associated with PUFA were *Streptococcus*, *Staphylococcus* and *Lactobacillus*. In particular, linoleic acid was significantly positively correlated with the abundance of *Staphylococcus* (*p* < 0.05), *Cupriavidus* (*p* < 0.05) and *Carnobacterium* (*p* < 0.05), suggesting that these bacteria promoted the production and accumulation of linoleic acid.

For fungi, the correlation network between major fatty acids and fungi was simpler than that of the bacteria, with only a few fungi showing significant correlations (Figure 6C). The main fungal community correlated with SFA were *Bullera* and *Candida*. *Bullera* was significantly negatively correlated with palmitic acid (*p* < 0.01) and positively correlated with stearic acid (*p* < 0.05), and *Candida* showed a positive correlation with stearic acid (*p* < 0.05). MUFA was correlated with *Penicillium*, *Trichoderma* and *Candida*. Among them, palmitoleic acid was positively correlated with *Trichoderma* (*p* < 0.05), oleic acid was negatively correlated with *Candida*. PUFA was correlated with *Trichoderma*, *Bullera* abundance variation. Among them, linoleic acid showed significant positive correlation with *Trichoderma* (*p* < 0.01) and positive correlation with *Bullera* (*p* < 0.05). This suggests that the above fungi are likely to be involved in the lipid metabolism process, and that they play an important role in lipid hydrolysis and the oxidation process.

### 3.9. Sensory evaluation

Descriptive analysis was carried out by trained panelists in order to gauge changes in the overall aroma profile of the bacon (Figure 6D). The sensory evaluation results were shown in Supplementary Table S5. Generally, the bacon sensory score gradually increased, with the highest score of 95.3 in the finished meat, indicating that the sensory quality of the bacon became increasingly good with the extension of time. The meat color, taste, odor and tissue state of the bacon increased in score with the change in the production process, but the taste score was the lowest in the curing period (T30), which was attributed to the uneven distribution of NaCl in the inner and outer layers of meat during the curing period.

## 4. Discussion

Microbiota in fermented meat products is important for the flavor formation of the final product. In this study, the dynamics of microbial succession, physicochemical parameters, and flavor substances during the natural fermentation of Longxi bacon, as well as their interrelationships were studied.

Microbial growth is highly correlated with the physicochemical conditions of fermented meat (Carmen et al., 2018). The physicochemical conditions affecting the flavor of Longxi bacon included NaCl, Aw, pH and amino acid nitrogen. In this study, Aw gradually decreased with the extension of fermentation time until the end of fermentation. Aw seemed to be one of the most important factors affecting the microbial community succession during the processing of Longxi bacon, and was closely related to the microbial growth and propagation, which is consistent with previous studies reported (Zhang M. et al., 2021). This study showed that Aw was positively correlated with the dominant microbial genera (*Psychrobacter*, *Cupriavidus*, *Carnobacterium*, *Phoma*, *Guehomyces* and *Bullera*) and significantly positively correlated with the non-dominant microbial genera (*Streptococcus* and *Lactococcus*). NaCl is another important factor affecting the microenvironment of bacon (Aaslyng et al., 2014). On the one hand, NaCl may cause physiological toxicity to microorganisms. On the other hand, it may create a highly permeable microenvironment that disrupts the electrolyte balance of the microorganisms (Gan et al., 2021). This may be a key factor for the lower microbial abundance and diversity during curing (curing stages I and II) than other processing periods. With the extension of fermentation time, NaCl concentration gradually increased and salt-tolerant microorganisms dominated, and the abundance and diversity of bacterial community increased in the air-drying stage. There were significant associations with dominant microbial genera (*Pseudomonas*, *Brochothrix*, *Trichoderma* and *Cryptococcus*) and non-dominant microbial genera (*Rhodotorula*, *Acremonium*, *Malassezia*, *Morchella*, *Trametes*, *Volvariella* and most bacteria). Amino acid nitrogen and pH were important factors affecting microbial growth in fermented meat (Tabanelli et al., 2012; Guan et al., 2022). RDA analysis showed that most microorganisms had no significant correlation with amino acid nitrogen and pH. Physicochemical results showed that amino acid nitrogen (from 0.176 mg/100 g in raw meat to 0.241 mg/100 g in bacon) and pH (maintained between 6.0 and 6.5) remained relatively during fermentation, which may be the main reason for the insignificant correlation between microbial abundance and amino acid nitrogen/pH.

Our work confirmed the microbial diversity and obvious microbial succession in the traditional Longxi bacon processing. The dominant bacteria in Longxi bacon samples may vary at different stages, but *Brochothrix*, *Carnobacterium*, *Pseudoalteromonas*, *Psychrobacter* and *Cupriavidus* were the predominant bacterial genera, which is consistent with the results of previous studies on smoked bacon. Meanwhile, HTS analysis showed that the dominant fungal genera of Longxi bacon were *Trichoderma*, *Phoma*, *Guehomyces*, *Cryptococcus* and *Bullera*, which differed from traditional fermented meat products (Zhang M. et al., 2021; Song et al., 2022). This may be attributed to differences in manufacturing processes (curing and air-drying) and physicochemical factors (pH, Aw, NaCl, temperature, osmotic pressure and nutrients) in addition to raw materials.

The flavor formation process of traditional bacon products is extremely complex. After the curing, air-drying (or smoking) and other processes, the resulting alcohols, acids, esters, aldehydes, ketones, hydrocarbons, terpenes, sugars, nucleotides, peptides and other substances complement each other to form the unique flavor base of bacon (Mottram, 1998). This study showed that aldehydes were the most abundant flavor components, especially hexanal, 3-methylbutyraldehyde and 2-octenal, which had low sensory thresholds and contributed more to the flavor. In addition, esters contribute considerably to the flavor of Longxi bacon. Esters have a unique fruity and wine-like aroma, and they have been detected in Suan zuo rou (Wang H. et al., 2021) and dry-cured hams (Sandra et al., 2018). The Longxi bacon esters are mainly ethyl octanoate and ethyl acetate, which are similar to the ester composition reported in the literature on most bacon products. Together with alcohols, such as linalool, ketones, olefins, furans and other substances, these form the Longxi bacon flavor system.

The formation of flavor substances in traditional fermented meat products is closely linked to microbial growth and metabolism. *Psychrobacter* and *Pseudoalteromonas* are the dominant bacteria commonly found in low temperature fermented foods, and both are significantly associated with the formation of ethyl acetate (Broekaert et al., 2013). *Lactobacillus* are the most widely used meat fermenters, its main functions are assimilation of carbohydrates to produce organic acids and catabolism of proteins, which play an important role in the flavor formation of fermented meat (Fontana et al., 2016). Olefins and aldehydes were also found to be the most abundant compounds in our study from the late curing to mid-air-drying period, and the highest abundance of *Lactobacillus* was found in bacon samples during this period, suggesting that the growth and metabolism of *Lactobacillus* are important for the formation of styrene and 3-methylbutyraldehyde. *Staphylococcus* is a common fermenting agent for meat fermentation, and its role in flavor production has been extensively studied (Fonseca et al., 2013). It can promote the β-oxidation process of fat to generate hexanal, valeraldehyde and octanal, thereby increasing the waxy flavor of sausages (Dalmis and Soyer, 2008; Gan et al., 2021). Our study also found that *Staphylococcus* had the highest abundance during the curing period (T15–T30), and aldehydes were mainly formed during the late curing and mid air-drying period, indicating that *Staphylococcus* has a certain influence on the formation of aldehydes. The main function of *Micrococcus* is to promote the peroxidative decomposition of substances, and its massive proliferation can increase the content of amino acids and short-chain fatty acids, which is conducive to the formation of bacon flavor (Papamanoli et al., 2002). The good correlation between *Micrococcus* and 2,3-octanedione in this study suggests that the growth and proliferation of *Micrococcus* has an important contribution to 2,3-octanedione production.

For fungi, mold is rich in amylase, protease and lipase systems, which have a strong catabolic effect on the three major nutrients and can produce special flavor substances (Scaramuzza et al., 2015). Yeast improves product flavor primarily by decomposing fats and proteins to produce peptides, phenols and alcohols (Bolumar et al., 2003). The flavor substances clearly associated with mold in this study were heptanal, 2-octenal and ethyl acetate. Yeast was involved in the formation of nonanal and ethyl acetate.

In conclusion, the bacon microenvironment (Aw, NaCl, amino acid nitrogen and pH) significantly influenced microbial community composition and succession, which was a key factor triggering the changes in microbial community. In addition, changes in food substrates and processing environmental conditions are also driving forces of microbial community succession. In fact, the microenvironment does not affect the microbial community alone, but rather interfere with the composition and succession of the microbial community in bacon samples through extremely complex synergistic effects.

## 5. Conclusion

In this study, we found the significant relationships between microbial community and quality factors (physicochemical properties, volatile compounds and fatty acids) during the fermentation of traditional Longxi bacon. In general, flavor formation is the result of a combination of bacteria and fungi. This study will improve our understanding of the fermentation mechanism in Longxi bacon production. Next, we will select a core group of functional microbiota for further analysis based on the correlation between microbiota abundance and the spectrum of the five flavor compounds, which will lay a foundation for further research on the food ecology of bacon.

## Data availability statement

The original contributions presented in the study are included in the article/Supplementary material, further inquiries can be directed to the corresponding author.

## Author contributions

YQ and JY contributed the experimental design, performed the statistical analysis, and wrote the manuscript. YQ, JY, YL, DA, and WZ contributed to manuscript revision, read, and approved the submitted version. All authors contributed to the article and approved the submitted version.

## Funding

The work was financially supported by the National Key Research and Development Program of China (2018YFD0400205).

## Conflict of interest

The authors declare that the research was conducted in the absence of any commercial or financial relationships that could be construed as a potential conflict of interest.

## Publisher’s note

All claims expressed in this article are solely those of the authors and do not necessarily represent those of their affiliated organizations, or those of the publisher, the editors and the reviewers. Any product that may be evaluated in this article, or claim that may be made by its manufacturer, is not guaranteed or endorsed by the publisher.
