# Supplementary material for: Microbial succession and its correlation with the dynamics of flavor compounds involved in the fermentation of Longxi bacon
Source: Front Microbiol. 2023 Aug 31;14:1234797. doi: 10.3389/fmicb.2023.1234797 (PMC10500841; doi:10.3389/fmicb.2023.1234797)
Supplement: Supplementary file 1 [file Data_Sheet_1.docx]

**Microbial succession and its correlation with the dynamics of flavor compounds involved in the fermentation of Longxi bacon**

Yuling Qu^1^, Jianmin Yun^1,^*, Yanhu Li^2^, Duiyuan Ai^1^, Wenwei Zhang^1^

^1^ College of Food Science and Engineering, Gansu Agricultural University, Lanzhou, China

^2^ Zhuanglang County Food and Drug Inspection and Testing Centre, Pingliang, China

***Corresponding authors:**

Jianmin Yun

E-mail: [yunjianmin@gsau.edu.cn](mailto:yunjianmin@gsau.edu.cn)

**Supplementary materials**

Supplementary Figure 1. Venn diagrams visualizes the similarity and difference of (A) bacteria and (B) fungi diversity in bacon at the different fermentation stages. Succession analysis of (C) bacteria and (D) fungi community in Longxi bacon at different fermentation stages.


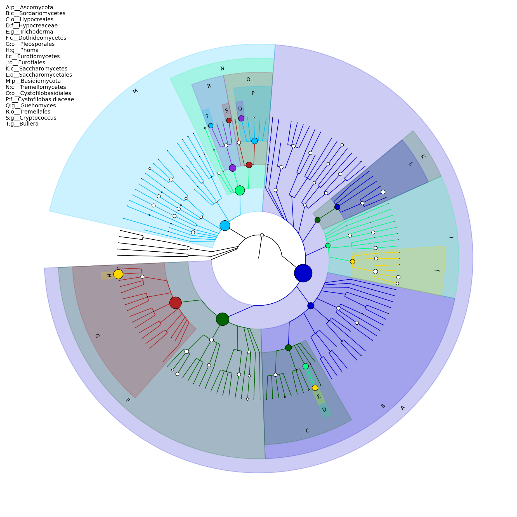


**D**


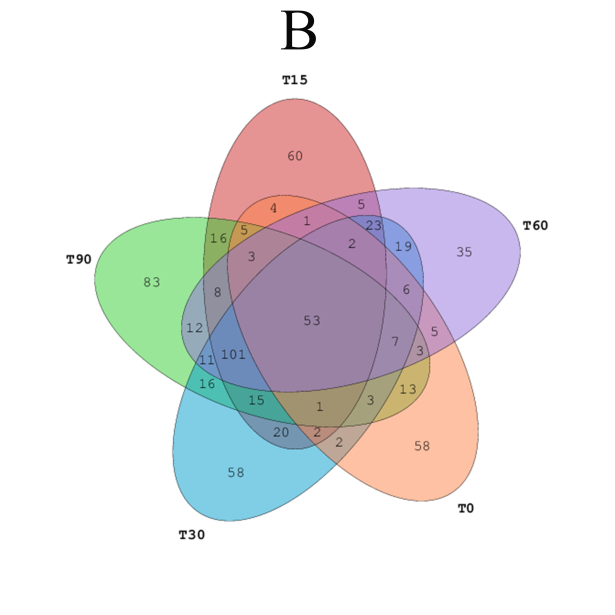


**B**


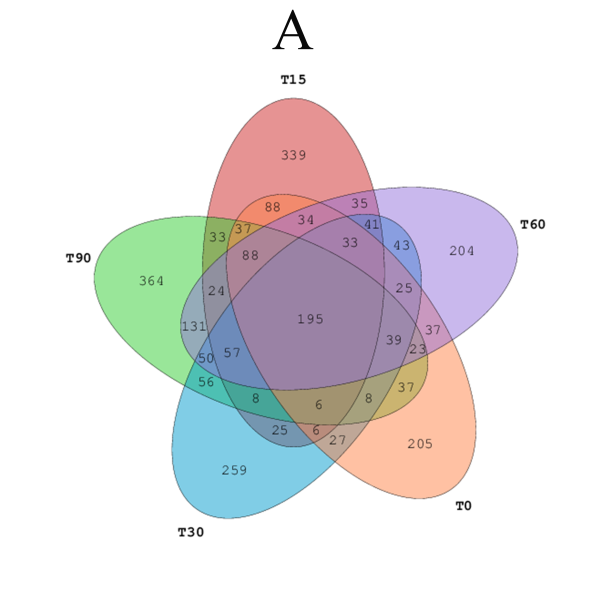


**A**


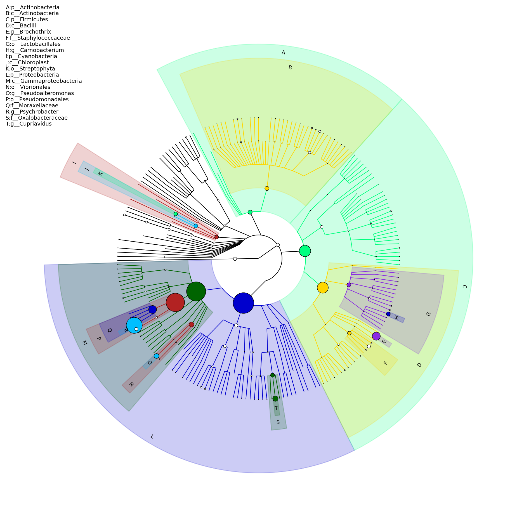


**C**

Supplementary Table 1. Sample information.

| Sample ID | number | Sampling period |
| --- | --- | --- |
| Raw meat | T0 | Raw meat stage (0^th^ d) |
| Cured stage I | T15 | the mid-stage of curing (15^th^ d) |
| Cured stage II | T30 | the post-stage of curing (30^th^ d) |
| Air-drying stage | T60 | the mid-stage of air-drying (60^th^ d) |
| Longxi bacon stage | T90 | Longxi bacon stage (90^th^ d) |

Supplementary Table 2. Alpha diversity indexes for 16S rDNA and ITS sequencing of the bacon samples.

| Organism | Sample ID | Reads | OTUs | Goods coverage | Chao1 | ACE | Simpson | Shannon |
| --- | --- | --- | --- | --- | --- | --- | --- | --- |
| Bacteria | T0 | 32940 | 106 | 0.992 | 888.00 | 888.00 | 0.946 | 6.11 |
|  | T15 | 37579 | 110 | 0.999 | 1051.45 | 1069.86 | 0.863 | 5.56 |
|  | T30 | 38421 | 128 | 0.996 | 885.84 | 909.28 | 0.925 | 5.92 |
|  | T60 | 37418 | 127 | 0.994 | 1059.26 | 1065.51 | 0.931 | 6.21 |
|  | T90 | 40508 | 109 | 0.999 | 1157.01 | 1169.47 | 0.954 | 6.53 |
| Fungi | T0 | 45680 | 139 | 0.978 | 168.94 | 170.94 | 0.89 | 4.67 |
|  | T15 | 38400 | 283 | 0.989 | 319.00 | 319.00 | 0.915 | 4.54 |
|  | T30 | 38868 | 307 | 0.978 | 339.00 | 339.00 | 0.91 | 4.67 |
|  | T60 | 44406 | 264 | 0.986 | 294.47 | 297.11 | 0.805 | 3.89 |
|  | T90 | 44980 | 317 | 0.984 | 351.82 | 355.96 | 0.899 | 4.75 |

Supplementary Table 3. Determination of key volatile flavor compounds in Longxi bacon.

| **Number** | **Compounds** | **Content（μg/kg）** | | | | |
| --- | --- | --- | --- | --- | --- | --- |
|  |  | T0 | T15 | T30 | T60 | T90 |
| **Hydrocarbons** | | | | | | |
| A1 | Styrene | 280.76 | 266.5 | 403.41 | 84.66 | 69.25 |
| A2 | D-Limonene | 2.51 | 272.53 | 160.98 | 15.6 | 84.61 |
| A3 | Lauricene | 0 | 68.54 | 72.67 | 8.19 | 55.22 |
| A4 | β-Phellandrene | 0 | 48.89 | 128.68 | 0.86 | 27.88 |
| A5 | β-Pinene | 0 | 10.05 | 31.29 | 14.9 | 83.54 |
| A6 | α-Pinene | 0 | 45.51 | 22.1 | 14.9 | 19.03 |
| A7 | 3-Carene | 0 | 46.88 | 3.03 | 11.16 | 5.09 |
| A8 | Terpinolene | 1.3 | 63.33 | 34.21 | 51.96 | 17.16 |
| A9 | 4-Phenyl-1-butene | 0 | 0 | 3.53 | 0 | 1.07 |
| A10 | β-Pinene | 0 | 43.14 | 51.07 | 28.71 | 10.81 |
| A11 | Dodecane | 8.15 | 0 | 0 | 10.53 | 0 |
| A12 | Tetradecane | 1.56 | 0 | 1.51 | 7.02 | 2.77 |
| A13 | 1,1-Dimethyl-2-octyl-cyclobutane | 2.25 | 3.47 | 2.52 | 2.89 | 0 |
| **Aldehydes** | | | | | | |
| B1 | (E,E)-2,4-heptadienal | 2.86 | 4.02 | 32.2 | 12.33 | 25.64 |
| B2 | (E,E)-2,4-nonadienal | 3.56 | 4.57 | 84.48 | 4.99 | 14.83 |
| B3 | 10-Octadecanal | 0 | 15.08 | 0 | 1.87 | 5.09 |
| B4 | 2,4-Decadienal | 14.91 | 4.39 | 116.67 | 13.89 | 13.94 |
| B5 | 2,4-Dodecadienal | 2.69 | 9.41 | 10.8 | 0 | 0 |
| B6 | 2-Heptenal | 18.12 | 6.12 | 152.81 | 52.67 | 34.4 |
| B7 | 2-Decenal | 14.83 | 1.28 | 38.15 | 2.96 | 9.83 |
| B8 | 2-Hexenal | 1.3 | 1.65 | 7.07 | 14.12 | 8.85 |
| B9 | 2-nonenal | 14.31 | 2.74 | 34.01 | 0.86 | 6.25 |
| B10 | 2-undecenal | 5.12 | 0 | 17.86 | 14.82 | 4.65 |
| B11 | 2-octenal | 179.14 | 61.6 | 18.27 | 9.05 | 55.4 |
| B12 | 3-Methylbutyraldehyde | 27.23 | 5.21 | 8.98 | 34.95 | 57.63 |
| B13 | Benzaldehyde | 1.17 | 6.31 | 6.96 | 9.52 | 32.08 |
| B14 | anisaldehyde | 0 | 41.86 | 105.98 | 3.51 | 58.35 |
| B15 | Heptanal | 4.34 | 0 | 9.49 | 9.52 | 5.72 |
| B16 | Adipaldehyde | 211.31 | 408.06 | 568.03 | 250.06 | 227.31 |
| B17 | Nonyl aldehyde | 32.95 | 15.81 | 96.59 | 67.8 | 135.37 |
| B18 | Dodecanal | 0.95 | 1.28 | 3.73 | 7.02 | 0 |
| B19 | Octanal | 144.98 | 93.04 | 157.85 | 20.36 | 104.36 |
| **Esters** | | | | | | |
| C1 | 4-Pinoresin acetate | 0 | 2.83 | 2.73 | 14.43 | 2.06 |
| C2 | Allyl butyrate | 1.65 | 4.84 | 1.31 | 1.01 | 15.37 |
| C3 | Ethylene caproate | 1.47 | 4.94 | 42.39 | 2.65 | 26.54 |
| C4 | Ethyl caproate | 25.23 | 3.29 | 48.14 | 37.92 | 70.05 |
| C5 | Linalyl formate | 0 | 56.21 | 16.45 | 2.26 | 2.59 |
| C6 | Ethyl Hexadecanoate | 3.99 | 0 | 0 | 1.09 | 1.16 |
| C7 | Ethyl caprylate | 23.5 | 106.47 | 21.7 | 49.39 | 31.72 |
| C8 | Linalyl acetate | 0 | 6.76 | 10.9 | 3.75 | 5.27 |
| C9 | Pine Oil Acetate | 0 | 46.98 | 21.2 | 0.78 | 6.79 |
| C10 | Geranyl acetate | 0 | 34.09 | 14.13 | 13.97 | 3.75 |
| C11 | Ethyl acetate | 11.53 | 1.74 | 6.96 | 1.56 | 101.59 |
| **Alcohols** | | | | | | |
| D1 | 1-Octen-3-ol | 13.7 | 5.67 | 66.21 | 20.36 | 23.32 |
| D2 | 2,3-Dibutanol | 0.95 | 2.74 | 1.92 | 8.58 | 26.54 |
| D3 | 2-Methyl-1-hexadecanol | 0 | 1.28 | 23.82 | 0 | 2.41 |
| D4 | 2-hexadecanol | 5.9 | 5.3 | 1.82 | 1.79 | 1.7 |
| D5 | 2-octen-1-ol | 2.6 | 0 | 9.39 | 2.5 | 10.81 |
| D6 | 4-Terpineol | 2.34 | 1.65 | 115.46 | 117.74 | 114.37 |
| D7 | 6-Perillyl alcohol | 0 | 17.27 | 5.65 | 0 | 14.65 |
| D8 | Eucalyptol | 0 | 65.98 | 181.67 | 165.02 | 178.34 |
| D9 | Phenylethanol | 9.8 | 16.45 | 21.4 | 16.7 | 19.12 |
| D10 | trans-2-undecen-1-ol | 6.59 | 1.19 | 4.44 | 2.34 | 2.86 |
| D11 | Linalool | 0 | 208.74 | 391.1 | 342.99 | 304.68 |
| D12 | Heptanol | 3.29 | 6.58 | 4.34 | 6.79 | 4.2 |
| D13 | Hexanol | 0 | 18.37 | 5.85 | 9.83 | 4.56 |
| D14 | Pinoresinol | 0 | 19.47 | 42.09 | 26.29 | 30.83 |
| D15 | Amyl alcohol | 4.51 | 0 | 12.82 | 1.72 | 4.65 |
| **Acids** | | | | | | |
| E1 | 2-Hydroxy-2-octyldecanedioic acid | 2.17 | 0 | 7.67 | 3.04 | 0 |
| E2 | 9-Hexadecenoic acid | 5.12 | 0 | 5.15 | 0 | 3.48 |
| E3 | Butyric acid | 3.47 | 1.01 | 24.12 | 1.17 | 0.98 |
| E4 | Heptanoic acid | 1.13 | 4.66 | 15.24 | 16.31 | 13.67 |
| E5 | Decanoic acid | 19.34 | 7.49 | 29.47 | 108.53 | 132.24 |
| E6 | Caproic acid | 28.09 | 16.18 | 144.83 | 59.3 | 111.33 |
| E7 | Nonanoic acid | 1.39 | 6.12 | 12.21 | 7.72 | 13.4 |
| E8 | Myristic acid | 13.44 | 6.03 | 12.72 | 24.19 | 27.25 |
| E9 | Octanoic acid | 9.88 | 10.97 | 30.08 | 71.24 | 84.79 |
| E10 | Lauric acid | 5.9 | 2.65 | 7.07 | 12.72 | 18.5 |
| E11 | 9-decenoic acid | 2.08 | 2.56 | 1.51 | 11.08 | 8.67 |
| **Ketones** | | | | | | |
| F1 | 1,13-Tetradecadien-3-one | 4.86 | 9.6 | 6.76 | 1.17 | 2.06 |
| F2 | Carvone | 0 | 9.6 | 3.03 | 1.72 | 0 |
| F3 | 2,3-Octanedione | 3.47 | 23.76 | 11.4 | 8.35 | 96.95 |
| F4 | 2-Pentanone | 1.13 | 36.83 | 1.21 | 33 | 2.14 |
| F5 | 2-octanone | 1.21 | 16.45 | 18.57 | 6.79 | 1.43 |
| F6 | 3-Methyl-6-(1-methylethyl)-2-cyclohexen-1-one | 0 | 9.87 | 16.05 | 0 | 14.56 |
| F7 | 3-Hydroxy-2-butanone | 0.95 | 62.79 | 430.97 | 1.25 | 2.32 |
| F8 | 3-Hydroxybutanone | 122.69 | 11.88 | 1.61 | 1.87 | 0.89 |
| F9 | acetophenone | 36.24 | 11.06 | 92.55 | 36.28 | 101.14 |
| F10 | Geranylacetone | 0 | 1.37 | 1.31 | 0.94 | 1.97 |
| **Other compounds** | | | | | | |
| G1 | p-Cresol | 3.29 | 0 | 11.1 | 19.19 | 18.14 |
| G2 | 4-Isopropyltoluene | 0 | 0 | 15.95 | 7.8 | 1.25 |
| G3 | 2-Pentylfuran | 3.47 | 1.19 | 13.42 | 2.18 | 18.23 |
| G4 | 4-Allyl anisole | 0 | 0 | 89.02 | 12.48 | 12.51 |
| G5 | Anisole | 4.25 | 169.07 | 23.62 | 175.55 | 69.34 |

Supplementary Table 4. Changes of free fatty acids in the production of Longxi bacon.

| Fatty acid types | | Content (%) | | | | |
| --- | --- | --- | --- | --- | --- | --- |
|  |  | T0 | T15 | T30 | T60 | T90 |
| Saturated fatty acids  （SFA） | Hexanoic acid (C6:0) | 0.06 | 0.01 | 0.01 | N.D. | N.D. |
|  | Heptanoic acid (C7:0) | 0.01 | 0.07 | N.D. | N.D. | N.D. |
|  | Octanoic acid (C8:0) | 0.15 | 0.02 | 0.03 | 0.01 | 0.01 |
|  | Nonanoic acid (C9:0) | 0.47 | 0.06 | 0.01 | N.D. | N.D. |
|  | Decanoic acid (C10:0) | 0.15 | 0.17 | 0.15 | N.D. | 0.09 |
|  | Lauric acid (C12:0) | 0.34 | 0.15 | 0.12 | 0.09 | 0.07 |
|  | Myristic acid (C14:0) | 2.77 | 1.45 | 2.5 | 1.55 | 1.43 |
|  | Pentadecanoic acid (C15:0) | 0.08 | 0.07 | 0.03 | 0.04 | N.D. |
|  | Palmitic acid (C16:0) | 25.41 | 16.92 | 20.58 | 20.5 | 22.06 |
|  | Heptadecanoic acid (C17:0) | 0.61 | 0.53 | 0.08 | 0.13 | N.D. |
|  | Stearic acid (C18:0) | 9.75 | 19.37 | 13.56 | 12.84 | 12.7 |
|  | Peanut acid (C20:0) | N.D. | 0.2 | 0.07 | 0.08 | N.D. |
|  | ΣSFA | 39.80 | 39.02 | 37.14 | 35.24 | 36.36 |
| Monounsaturated fatty acids（MUFA） | Methyl heptanoate (C7:1) | 0.37 | 0.34 | 0.12 | 0.18 | N.D. |
|  | Tetradecylenic acid (C1:1) | 0.06 | 0.03 | 0.02 | 0.03 | 0.02 |
|  | palmitoleic acid (C16:1n7) | 7.5 | 2.36 | 5.1 | 3.91 | 2.84 |
|  | Heptadecenoic acid (C17:1) | N.D. | 0.17 | N.D. | N.D. | 0.06 |
|  | Oleic acid (C18:1n9c) | 37.31 | 30.91 | 40.46 | 39.84 | 38.16 |
|  | Eicosatetraenoic acid (C20:1) | 0.77 | 1.62 | 1.17 | 0.6 | 0.17 |
|  | ΣMUFA | 46.01 | 35.43 | 46.87 | 44.56 | 41.25 |
| Polyunsaturated fatty acids（PUFA） | Methyl linoleate (C18:2n6c) | 3.87 | 15.73 | 12.73 | 13.04 | 14.62 |
|  | α-linolenic acid (C18:3n6) | N.D. | 1.23 | 0.66 | N.D. | 0.38 |
|  | γ-linolenic acid (C18:3) | 6.36 | N.D. | N.D. | N.D. | N.D. |
|  | Eicosadienoic acid (C20:2) | N.D. | 1.82 | 0.32 | 0.41 | N.D. |
|  | Eicosatrienoic acid (C20:3n3) | 0.09 | 0.23 | 0.08 | 0.24 | 0.15 |
|  | Eicosatrienoic acid (C20:3n6) | N.D. | 0.19 | N.D. | N.D. | N.D. |
|  | Arachidonic acid (C20:4n6) | 0.97 | 0.22 | 0.63 | 1.91 | 1.62 |
|  | Docosapentaenoic acid (C22:5n3) | 0.15 | 0.1 | 0.12 | 0.25 | 0.15 |
|  | Docosapentaenoic acid (C22:4) | 0.13 | 0.19 | 0.27 | 0.27 | N.D. |
|  | ΣPUFA | 11.57 | 19.71 | 14.81 | 16.12 | 16.92 |
| ΣPUFA/ΣSFA | | 0.291 | 0.505 | 0.399 | 0.457 | 0.465 |

Supplementary Table 5. Sensory evaluation table of Longxi bacon production.

| Sensory evaluation | | | | | | |
| --- | --- | --- | --- | --- | --- | --- |
| Sample | Flesh color  (20%) | Mouthfeel (20%) | Odor  (20%) | Taste  (20%) | Tissue condition (20%) | Total score |
| T0 | 16.2±0.92^b^ | 16.1±0.99^d^ | 16.7±1.25^c^ | 17.6±1.17^bc^ | 16.6±0.97^c^ | 83.2±5.3^d^ |
| T15 | 17.1±0.99^b^ | 17.3±0.67^c^ | 17.4±0.70^c^ | 17.3±0.67^c^ | 17.4±1.07^bc^ | 86.5±4.12^c^ |
| T30 | 18.1±1.29^a^ | 18.0±1.25b^c^ | 18.2±0.92^b^ | 17.5±1.27^bc^ | 17.8±1.03^b^ | 89.6±5.75^b^ |
| T60 | 18.6±0.52^a^ | 18.5±0.53^ab^ | 18.8±0.42^ab^ | 18.2±0.42^b^ | 18.7±0.82^a^ | 92.8±2.71^a^ |
| T90 | 18.7±1.34^a^ | 19.2±0.79^a^ | 19.1±0.88^a^ | 19.4±0.70^a^ | 18.9±0.99^a^ | 95.3±4.70^a^ |
